# Brief encounters: what do primary care professionals contribute to peoples’ self-care support network for long-term conditions? A mixed methods study

**DOI:** 10.1186/s12875-016-0417-z

**Published:** 2016-02-17

**Authors:** Anne Rogers, Ivaylo Vassilev, Helen Brooks, Anne Kennedy, Christian Blickem

**Affiliations:** NIHR CLAHRC Wessex, Faculty of Health Sciences, University of Southampton, Building 67, Highfield Campus, University Road, S017 1BJ Hampshire, UK; NIHR CLARHC Greater Manchester, School of Nursing & Midwifery University of Manchester, Williamson Building, M139PL Manchester, UK; Centre for Public Health, Liverpool John Moores University, Liverpool, L3 2AJ UK

**Keywords:** Primary-care professionals, Long term conditions, Social networks, Self-management support, Mixed methods

## Abstract

**Background:**

Primary care professionals are presumed to play a central role in delivering long-term condition management. However the value of their contribution relative to other sources of support in the life worlds of patients has been less acknowledged. Here we explore the value of primary care professionals in people’s personal communities of support for long-term condition management.

**Methods:**

A mixed methods survey with nested qualitative study designed to identify relationships and social network member’s (SNM) contributions to the support work of managing a long-term condition conducted in 2010 in the North West of England. Through engagement with a concentric circles diagram three hundred participants identified 2544 network members who contributed to illness management.

**Results:**

The results demonstrated how primary care professionals are involved relative to others in ongoing self-care management. Primary care professionals constituted 15.5 % of overall network members involved in chronic illness work. Their contribution was identified as being related to illness specific work providing less in terms of emotional work than close family members or pets and little to everyday work. The qualitative accounts suggested that primary care professionals are valued mainly for access to medication and nurses for informational and monitoring activities. Overall primary care is perceived as providing less input in terms of extended self-management support than the current literature on policy and practice suggests. Thus primary care professionals can be described as providing ‘minimally provided support’. This sense of a ‘minimally’ provided input reinforces limited expectations and value about what primary care professionals can provide in terms of support for long-term condition management.

**Conclusions:**

Primary care was perceived as having an essential but limited role in making a contribution to support work for long-term conditions. This coalesces with evidence of a restricted capacity of primary care to take on the work load of self-management support work. There is a need to prioritise exploring the means by which extended self-care support could be enhanced out-with primary care. Central to this is building a system capable of engaging network capacity to mobilise resources for self-management support from open settings and the broader community.

**Electronic supplementary material:**

The online version of this article (doi:10.1186/s12875-016-0417-z) contains supplementary material, which is available to authorized users.

## Background

Globally the importance of self-management has been acknowledged as relevant for maintaining and improving the quality of life of people living with a long-term condition. Self-management support (SMS) interventions of new technologies, information provision, skills training and support from health professionals have been associated with increased patient empowerment and self-efficacy, changes in behaviour and a reduction in the utilisation of healthcare resources [1]. Providing SMS support on an ongoing bases involves a wide set of relationships, resources in managing health risks over time and the ability to plan for and respond to new management needs [1–3]. Primary care and general practice have been identified as having pivotal roles to play in supporting long term condition self-management based on the assumption of their organsiational capacity to offer, ready access, relevant clinical information systems, decision support, person centredness, continuity of care and behavioural interventions [4–7]. However, alongside the aspiration of realising an expanded contribution to SMS questions have been asked about the capacity of general practice to deal with the complexity and intractability of chronicity presented by patients [8]. For example, there is evidence, of the failure of self-management topics being treated as legitimate objects for discussion within consultations or framed in a way which reinforces medical agendas at the expense of patient initiated self-management concerns [9–11]. Moreover, primary health care professionals have been found to prioritise retaining control over referral and disease management and downgrading the need for connections with self-management support arrangements from external agencies [12–14].

In recognition of these limitations some have advocated extending personalised care planning in primary care in a way which forms part of a wider connected up system of chronic illness management, commissioning and partnership working [15, 16]. However, despite a number of initiatives the capacity of primary care to provide this broad range of support remains open to question [17]. Studies in a UK context have indicated the existence of barriers to implemention [13] primacy given to ‘surveillance’ of patients with an insufficient capacity being made available for shared decision making amongst those most likely to benefit [10, 11, 18, 19]. A recent review pointed to the difficulties in implementing self-management support at the level of the consultation because of tensions around forming partnerships with patients and the control expected by professionals of patients over their illness [20]. Yen et al. [21, 22] point to evidence of the prioritisation of the importance of professional activities and those of other professionals to improve management of chronic illness *rather* than those of patients and carers. However, attempts to explore and engender a whole systems approach have to date done so in the absence of relatively little attention being given to the patient’s perspective of the value of self-management related activities, practices and outcomes in primary care that makes a contribution *relative* to those made by others from within a personal network of support.

In this paper we map the nature and type of work undertaken by people in the personal networks of those with a long-term condition in order to identify the relative input and nature of the perceived role of primary care professionals relative to other social network members. In order to explore the value of primary care within a broader set of personal ties we have used a social-network approach to making sense of how long term illness support, and relationships are implicated in the mobilisation and use of resources for long term condition [23]. We use the notion of ‘personal community’ to refer to a group of people who contribute to an individual’s well- being through providing support and approval. In the context of SMS a personal community is conceptualised as a self-management workforce providing condition relevant support consisting of personal ties characterised by certain network properties (network size, density, degree of fragmentation).

## Method

A cross-sectional mixed methods study was conducted between April 2010 and January 2011 incorporating a postal questionnaire and a face-to-face network interview (the full description of study design is reported elsewhere) [23].

### Quantitative survey

2001 patients with chronic heart disease (CHD) or diabetes were randomly selected from the disease registers of consenting GP practices in deprived areas of the NW of England and sent invitation letters. A total of 300 people responded to invitation letters and completed both elements of the study. Data on network members was captured and mapped using the method of “concentric circles of importance”. Involvement was identified through identifying and describing the members who make up the personal communities of individuals and how they were valued in importance combined with the illness ‘work’ undertaken, understood as the contribution of network members to various activities [encompassed under three domains: illness specific, everyday and emotional work].

- ***Illness specific work*** is concerned with: taking medications; regimens of taking and interpreting measurements; understanding symptoms; making appointments.
- ***Everyday work***: the tasks of housekeeping and repairing; occupational labour; child rearing; support and activities related to diet and exercise, general shopping and personal care.
- ***Emotional work*:** work related to comforting when worried or anxious, everyday matters, including health, well-being and companionship. It includes a biographical dimension associated with the reassessment of personal expectations, capabilities and future plans, personal identity, relationships and biographical events.

We produced questions relating to each category of work to capture the role of different network members from the perspective of the individual. For the purposes of the analysis categories were combined. During the interviews, participants were asked to elaborate on the roles of network members by rating each between 1 and 5 on a Likert scale for 17 different aspects of work undertaken by members where 1 is ‘not at all’ and 5 is ‘a lot’. In addition data was collected that measured the types of relationship present in each person’s network: the *perceived* closeness of network members, the size of network and the fragmentation and density associated with individual networks [24]. We worked with the notion that having a long-term condition requires different types of work by extending this from a focus on the individual to network members.

### Qualitative interviews

A semi-structured interview formed part of the survey to further explore the role of individualnetwork members, and the interview questions can be found in Additional file 1. The broad focus of the interview was on participants’ management of long-term health conditions (diabetes and chronic heart disease), the rationale for the placing and configuration of network members and descriptions and the values placed on social networks and relationships. The qualitative interviews allowed further elaboration of the meaning and contribution of relationships to networks and the nature of the illness work undertaken. The analysis was based on a purposively selected set of transcribed interviews of respondents who had identified differing contributions to illness work including those who had made reference to primary care professionals (and those who had not) within varying combinations of network types (i.e. those with only a professional and those where primary care formed part of a much broader spectrum of network member input). The response and attributions of value were made mainly in the dialogue and responsiveness to a statement introduced by the interviewer as part of a ranking exercise linked to the circle placement of a persons’ support network.

### Qualitative analysis

The interviews were transcribed verbatim and analyses assisted by Atlas (version 6). Aframework analysis was undertaken, coding data relating to the work (emotional, illness specific, or everyday) and positioning of social network members (from inner to outer circle). The researchers coded transcripts independently and then met to discuss, examine and agree on emergent codes. A list of final themes and related sub-themes were produced which related to the value attributed to primary care professionals and the work and input provided to self-management support.

All participants gave informed written consent to take part in the study. Ethical approval was obtained from the Greater Manchester Research Ethics Committee in February 2010 (ref: 10/H1008/1). All participants received £10 gift vouchers as a compensation for their time and effort.

## Results

### Quantitative results

#### Primary Care and Health professionals

Of the 300 respondents in the survey sample, 89.0 % (*n* = 267) placed one or more health professionals in their network. The 300 personal networks generated 2544 network members, (not including the respondents themselves). 600 (23.6 %) were health professionals 395 (two-thirds 67 %) were primary care professionals. In addition to being cited more frequently than secondary care professionals in making a contribution in terms of **relative** value primary care professionals were more likely to be viewed as being of more central importance than other professionals (e.g. those operating from secondary care). Just over half of the primary care professionals were placed in the central circle and just under half in the middle and outer circle. This compared to a third and two thirds respectively for professionals located in secondary care and elsewhere (Table 1).

### Multilevel linear regression

Table 2 summarises the mean amount of types of work undertaken by different categories of relationship type within networks. A number of statistically significant differences in mean work undertaken were found between primary care based professionals and other relationship types. Differences were significant (*p* < 0.001) for all work domains. Partners/spouses performed the most work in all three categories of work. They reported the highest mean work scores in the emotional, everyday and illness specific domains, followed by close family. Primary care professionals were rated as second lowest in terms of contribution to emotional work and everyday work, but were third with regard to their contribution to illness specific work

**Table 1:** Perceived importance as defined by circle location and type of relationship

Relationship type	Centre (*n*/%)	Middle (*n*/%)	Outer (*n*/%)	Total
Partner or spouse	165 (92.7 %)	11 (6.2 %)	2 (1.1 %)	178
Close family	483 (66.6 %)	191 (26.3 %)	51 (7.0 %)	725
Other family	96 (47.3 %)	92 (45.3 %)	15 (7.4 %)	203
Friends	140 (26.9 %)	265 (50.9 %)	116 (22.3 %)	521
Pets	35 (53.0 %)	15 (22.7 %)	16 (24.2 %)	66
Primary care professionals	211 (53.4 %)	125 (31.6 %)	59 (14.9 %)	395
Other healthcare professionals	65 (31.7 %)	87 (42.4 %)	53 (25.9 %)	205
Groups	36 (21.2 %)	59 (34.7 %)	75 (44.1 %)	170
Other	28 (34.6 %)	30 (37.0 %)	23 (28.4 %)	81

**Table 2:** Domain of self-care work by relationship category

	Number	Mean (SD)	
Emotional work			
Partner or spouse	178	7.85 (2.54)	
Close family	725	4.72 (3.03)	
Other family	203	4.04 (2.96)	
Pets	66	4.01 (2.91)	
Friends or colleagues	521	3.15 (2.72)	*p* < 0.001
Groups	170	2.76 (2.36)	
Other relationships^a^	81	2.25 (2.82)	
Primary care professionals	395	1.70 (2.17)	
Other healthcare professionals	205	1.13 (1.77)	
Total	2544	3.58 (3.15)	
Illness specific work			
Partner or spouse	178	6.47 (3.07)	
Close family	725	2.49 (2.55)	
Primary care professionals	395	2.44 (1.93)	
Other family	203	1.87 (2.36)	
Other healthcare professionals	205	1.77 (1.71)	*p* < 0.001
Other relationships^a^	81	1.44 (1.92)	
Friends or colleagues	521	1.22 (1.79)	
Groups	170	0.74 (1.08)	
Pets	66	0.66 (0.82)	
Total	2544	2.19 (2.53)	
Everyday work			
Partner or spouse	178	6.37 (2.97)	
Close family	725	1.67 (2.39)	
Pets	66	1.21 (1.56)	
Other family	203	1.03 (1.97)	
Primary care professionals	395	0.92 (1.55)	*p* < 0.001
Other relationships^a^	81	0.85 (1.56)	
Groups	170	0.77 (1.51)	
Friends or colleagues	521	0.71 (1.60)	
Other healthcare professionals	205	0.65 (1.40)	
Total	2544	1.46 (2.42)	
	N	Mean (SD)	

^a^Other relationships included carers, volunteers and food delivery service

Whilst half of all the identified primary care professionals were placed at the centre they were less likely than family members to be ‘superhelpers^1^’, i.e. network members who provided intense and wide ranging support (see Table 3).

**Table 3:** Network members who provide high amount and range of support (superhelpers) by relationship type

Relationship type	Superhelper (%/*n*)	Not Superhelper (%/*n*)
Partner or spouse	87.6 % (156)	12.4 % (22)
Close family	33.2 % (241)	66.8 % (484)
Other family	23.2 % (47)	76.8 % (156)
Primary care professionals	17.0 % (67)	83.0 % (328)
Friends	15.7 % (82)	84.3 % (439)
Pets	15.2 % (10)	84.8 % (56)
Other	9.9 % (8)	90.1 % (73)
Other healthcare professionals	8.8 % (18)	91.2 % (187)
Groups	5.9 % (10)	94.1 % (160)
Total	25.1 % (639)	74.9 % (1905)

### Qualitative results

We use qualitative data derived from the ranking of contribution of social network members to provide further illumination about what lay behind the quantitative results in terms of the content ranking and relative contribution made to SMS support by primary care professionals. How people made judgements about the contribution made and what was most valued about primary care was predicated firstly on the evaluation made about contributions of PCPs relative to their own actions others in their personal network and secondly on perceptions about what was on offer from the supply side of primary care.

#### Value ratings framed with reference to self and others and the supply side of primary care

The capacity to rely on one’s own self-action provided the framing of evaluating what others in a network provided. The GP and other primary health care professionals fitted around this sense of self with others in a network providing support.

- R: Well, once we’ve spoken about what I need to do, *it is me who deals with it*.
- I: Yeah. So, would you not really rate her on that?
- R: Not really, no. I’d say it’s a one.
- I: Yeah. And then, how about the GP?
- R: Same again, it’s a one, yeah, because it’s me who deals with it, it’s not them who deals with it, it’s me.
- I: And then, ‘This person helps me understand advice, so I know what I have to do to manage my condition’.
- R: I think I deal with that myself anyway.
  I know what I’ve got, and I know how to deal with it. (GP157).

Comparisons made about support and relationships with other network members revealed the relative value of support gained from primary care professionals. In networks with dense extended family networks primary care professionals were typically seen as a back-up in case things went seriously wrong. The quotes below suggest that the family may be in a position to help with the immediacy of management whilst the GP’s role is more distant and infrequent. It was not normally expected that GPs should get personally involved in everyday matters or how people felt:

- I: Right. If you were struggling, would you talk to anyone?
- R: If I was struggling, which one would I talk to? It would be my daughter **[H)**, the first port of call.

- R: …..The GP wouldn’t…to them I’m just another figure aren’t I?
- I: What would you score them?
- R: A four, quite high.
- I: …..GP and nurse?
- R: The nurse basically, I very rarely see my GP.
- I: Okay, well, shall we score the nurse?
- R: Probably four.
- I: And GP?
- R: Probably about three, I rarely see him. (GP082)

Primary care’s ideal portrayal as providing a comprehensive range of inputs to self-management support, was not in evidence from our respondents. At best there was fleeting mention of a more extensive role in chronic illness management and there were indications that primary care might not currently be the best place for such management.

…they’re only really just started to gather information like on diabetes. Because it’s a general practice and you go and see them when you’re sick basically, and you can go and see them for a specialist condition. (GP142)

However, there was a perception that elements of management previously undertaken by specialists in secondary care were being adopted by primary care which elevated the latters value.

- R: now the GP is more important because they’ve become…specialist in diabetes. Yeah, more of a health centre.
- I: Right.
- R It’s not like the big health centres that you see but the surgery has got a specialist area for diabetes.
- I: Okay, good.
- R: And they have an asthma clinic, and things like that, so they’re specialising, so you can go there and talk to someone who does know. (073)

A low level of input was not always a reason for not valuing a GP highly as long as access was guaranteed when assistance was requested or required. However there was little evidence of spontaneously viewing primary care as a place one asked for or received resources or assistance with lifestyle or information.

Well they normally send for me, maybe once a month for me to have a blood test. So I definitely go once a month, and I went again last week, because of this. I don’t go unless there’s something wrong, I don’t go just if there is something not right, I will make an appointment. (GP0123)

#### The essential value of primary care professionals: Access to medication as a restricted and regulated resource

Commensurate with previous research about patient experience of primary care ease of access to formal provision for higher level involvement when needed was valued highly. However it was medication that constituted the centrality of expectation and demand from GPs in terms of keeping a long-term condition under control on an ongoing everyday basis. The supply of medication through prescribing was frequently the reasons cited for GPs being placed in the central concentric circle (i.e. representing high value). In this respect primary care professionals were viewed as gatekeepers to a highly valued resource without which a long-term condition could not be managed and which others in a personal community could not provide. *he's (GP)the one who sorts me out like, you know, pill wise and stuff like that’*. (GP089) The importance and value of access to medication was expressed by this respondent who had little regard for his GP but for whom medication was considered to be important for his ability to manage on a daily basis:

- R:………that GP I've got, he's an absolute idiot.

- I: Okay, so would you put him this diagram as important for managing your health or not?
- R: Well he writes the prescriptions out so I suppose he's got to go on there, hasn't he?

Well he does help me, he writes prescriptions so you'd better put two (2^nd^circle) GP099

The high value placed on access to medication was distinguished from the task of prescribing. This was viewed as a fairly low level routine task and a limited role when seen as the main component of an overall valued contribution by the GP who in other ways was seen as disconnected from the problems and struggles of living with a long term condition.

- R: All the doctor does is they give me the prescription for me to get all my tablets that I'm on.
- So maybe one of the outer circles…
- I: Do you feel the GP helps you value and enjoy life would you say or …
- R: Well the GP has no part of me life at all except for the fact that they
- if I go to them when
- I’ve a problem they diagnose it and give me a prescription, that’s the beginning and the end of my relationship with my GP (381)

However when the work of medication was viewed as part of a wider approach to managing a long-term condition this was more highly valued.

- I: What would you give them on that?
- R: Well, I mean whatever I’m on is holding me nice and steady, so I’ve got it top marks I suppose, you know.
- I: Yeah. So would they be both…
- R: They do…like for instance, (they say) G, it’s a while back now, oh your blood pressure’s a bit high, we’d better just change that pill to that pill. And it come back down. You know what I mean, she’s tweaking it up and that, and keeping…
- I: So you’re happy with the way they do that?
- R: Yeah.
- I: So would you give them a five (highest value) on that?
- R: Yeah I would, yeah. (GP089)

#### Relational and informational support recognised as delegated work

The delegation to nurses of chronic illness work was evident and detectable in the value and location attributed to primary care professionals within the network by respondents. Chronic illness work incorporating communication trust and reciprocity were all viewed as having been downwardly delegated from GP’s to nurses. The increased role of nurses accounted for the attribution of a higher subjective appreciation of their input compared to GPs where minimalist and distant contact was more in evidence. Trust in the nurse is also seemingly shifted from the GP with the ongoing and more frequent contact accompanying less contact with the GP.

And how often would you see him, do you think?

- R: Him? I have a review every twelve months but, obviously, if there’s problems in between.
- I: Yeah. And do you tend to go in between, or?
- R: I usually go to my diabetic nurse. I’ve just changed one of my tablets now and I’ve done it through her, I’ve gone and told her and she’s gone to see the doctor.
- I: So he’s a bit less often than her?
- R: Yes.
- I: And how long would you spend with him when you do see him?
- R: Five minutes [laughs] (GP607)
- I: If you think about your diabetes and the different people and organisations that help you manage it, who would go in this middle **[nurse placed in 1st circle]**?
- R: Well it would be T **[practice nurse]**, because she is helping me with being diabetic, she takes all the blood from me.
- I: This is the nurse at the GP surgery?
- R: Yes, yes, yeah… called T. I’m there on Monday as a matter of fact. She does all my blood, my blood pressure, weighs me and everything, she’s smashing.
- I: Oh that’s good, anybody else go in that middle?
- R: Well there is only her really. My doctor doesn’t really, all the doctor does is they give me the prescription for me to get all my tablets that I’m on. (GP056)

Primary care nurses tended to be seen as more relational and less personally distant and thus trusted more in terms of advice and input about chronic illness.

- I: So the nurse, when you see the nurse, how long does it take?
- R: I don’t speak… I don’t speak to her. I go down there. So it’s whatever it needs. You can talk to her. She doesn’t fob you off or anything. You can… you can speak to her if you want to about anything. She always asks if I’m depressed? I say, not about the diabetes. I might be worried but it’s not about the diabetes. (GP082)

## Discussion

### Summary of findings

This study explored the value and input of primary care professionals within the personal networks of those with a long-term condition using a concentric circles method to identify the work and value attributed to social network members. Quantitative assessment explored the amount and type of work undertaken by primary care professionals relative to others in the network. Primary care professionals were more highly rated than other professionals (i.e. those in secondary care) but less so than others in the network such as close family or spouses). Partners and spouses provided most support in terms the amount and intensity of illness, emotional and every-day work. Most support provided in primary care is work related to illness specific management (medication etc.) whilst the provision of emotional support is lower than that obtained from other network members (family, spouses, friends, groups, pets) [22, 25, 26]. The intensity of support provided by primary care professionals was found to be t less tess that that recieved from other social network members.

The qualitative analysis was integrated with the quantitative in order to confirm, contrast and elaborate on these findings. Evaluations of the contributions of ‘others’ were made in terms of the extent to which what they offered reinforced the primacy of self-action in managing a condition [27]. The importance attributed to accessing prescribed medication as the principle value of primary care input reflects the importance attributed to self-medication-work as a highly personalised form of self-management undertaken by the individual and the limited offering of support from primary care. The descriptions provided by respondents as to what they perceived to be available from primary care was of minimalist support for illness management. This picture of minimal provision is in stark contrast to comparisons made with the elaborated picture painted of the recent and aspirational role expected from primary care from literature and policy discussions. The expectations of policy makers that the central pillars of traditional self-management support provided through a process of information-sharing, shared decision-making and action planning did not feature in the narratives of the participants of this study. There was little suggestion for example that respondents in this deprived community sample were able to access or offered a designated programme of chronic illness management or self-care support. Nor was there mention of the elaborated decision support, patient centred engagement or psychological support characterising trials of SMS that have recently been tested in primary care contexts [28]. Thus, primary care professionals can be described as providing ‘*minimally provided support*’.

However, there was little indication from the accounts of respondents that more self-care support was expected. This seemed to be related not only to a realistic evaluation of what was on offer from the supply side of General Practice but was linked to a reliance on resources of the self and those in the network. Accounts suggested that the greatest trust and focus on support rested with the individual and others in the social network. Whilst, claims to holism and emotional support that GPs claim is central to their role was not evident from accounts, primary care nurses were sometimes identified as providing informational and relational support and this was recognised as delegated work . The results presented here highlights the central focus on bio-medical management currently characterising primary care provision in relation to long-term conditions and the relative absence of self-management support. This points to the limitations of an over-reliance on the promise of expanding self-care support in primary care and the need to look beyond this in terms of engaging with open system resources accessible to people with long-term conditions in domestic and community settings [29, 30].

### Strength and limitations

The strength of the current study include the use of mixed methods to show the rationale behind the quantitative analysis of relative value. There are limitations to this research. Our case study focuses on people with type 2 diabetes and it may be that people with other long-term conditions view their support from primary care differently. However, given the primacy accorded to diabetes in national policies and guidelines and the high percentage within the population, we feel the findings provide an important insight into how self-care support is being enacted in primary care.

## Conclusion

Primary care professionals have an important and relevant place to play in the management of long-term conditions. The prescribing of medication and routine bio-medical monitoring constitutes a central element of the provision of support. Evidence of the increasing burden of bio-medical responsibilities in primary care combined with the desire to be in control and the low expectations of this deprived group of patients suggests that extended self-care support might not always be best mediated through the current GPs and primary care system Given the relatively low expectation and response from people with long-term conditions, primary care aspects of self-management support in the future might be orientated more to the way in which people directly connect to and access resources which are distributed beyond the confines of primary care but are relatively hidden such as in the voluntary sector. Other avenues of support might be more acceptable and appreciated if they were accessed independently from general practice. Individuals do not expect an extended role from primary care and seem ready to access information and other ways of managing everyday life themselves. The latter might be reinforced and facilitated by primary care but not directly managed by them.

Facilitating access to resources for enhancing self-management by agents and agencies which are not necessarily encompassed within the traditional organisational boundaries of primary care are likely to be be more worthwhile.

Voluntary and community organisations which allow for social involvement and linked to improved outcomes for self management support [31] are likely to enhance self-care support in the future. If such pathways are to be developed effectively resources for achieving this would need to be channelled into localities, local authorities, and community based organisations.

## Additional file

Additional file 1:
**Prompt questions for qualitative interviews.** (DOCX 14 kb)

## Footnotes

1 Super-helper was defined as a network member whose scores was equal or above the mean scores for each of the three types of work (illness specific, emotional, everyday).
