# Supplementary material for: Brief encounters: what do primary care professionals contribute to peoples’ self-care support network for long-term conditions? A mixed methods study
Source: BMC Fam Pract. 2016 Feb 17;17:21. doi: 10.1186/s12875-016-0417-z (PMC4756522; doi:10.1186/s12875-016-0417-z)
Supplement: Additional file 1: — Prompt questions for qualitative interviews. (DOCX 14 kb) [file 12875_2016_417_MOESM1_ESM.docx]

**Additional file 1**

Prompt questions for qualitative interviews

1. How do those in your diagram help you manage your condition day to day?

2. What do they do to help you cope with your illness?

3. Where or who do you go to find out more about your illness?

4. Is there anything else that you find useful to help you cope with your illness?

5. When you need advice about, or help with, your diet, who do you go to?

6. When you need advice about, or help with, exercise, who do you go to?

7. Where would you go, or who would you go to, for advice or help with relieving stress?

8. When you need advice about, or help with, medications who would you turn to?

9. a) Living with a long-term condition often means that you need to do things more slowly, take on additional tasks and other people may need to make compromises that are good for your health. Who in your diagram does these things?

b) Please describe in detail what would you do on a typical day starting from getting up in the morning. Please include tasks and activities that are not related to managing your condition, such as cooking, cleaning, making repairs, etc. Can you tell us how different people on your diagram are involved with different activities?

10. Who do you turn to when you are worried about your illness?

11. a) Looking at your diagram, who do you think you would like to be more involved in helping you with your illness than they are at present?

b) What and who helps or hinders your care (related to diet/exercise/medication)? Can you think of examples?

13. Who or what in your diagram gives you emotional support and encouragement? Can you think of examples?

14. Who in your diagram would step in/stand up for you when you do not feel well enough to stand up for yourself?

15. Who among those in your diagram do you help? How?
